# Autism Spectrum Disorder (ASD) and Attention Deficit Hyperactivity Disorder (ADHD) With Language Impairment Accompanied by Developmental Disability Caused by Forkhead Box Protein 1 (FOXP1) Exon Deletion: A Case Report

**DOI:** 10.7759/cureus.20595

**Published:** 2021-12-22

**Authors:** Shuliweeh Alenezi, Ahmed Alyahya, Hesham Aldhalaan

**Affiliations:** 1 Department of Psychiatry, College of Medicine, King Saud University, Riyadh, SAU; 2 Department of Psychiatry, Eradah Complex for Mental Health, Riyadh, SAU; 3 Neuroscience, King Faisal Hospital and Research Center, Riyadh, SAU

**Keywords:** genetic expression, foxp1, developmental disability, adhd, asd, case report

## Abstract

Forkhead box protein 1 (FOXP1) (OMIM: 605515) is located at chromosomal region 3p14.1, which codes for a transcriptional repressor protein. FOXP1 syndrome (FOXP1S) (OMIM #613670) is caused by FOXP1 gene deletions and mutations (nonsense, missense, and in-frame deletions). It is identified by the presence of intellectual disability with language impairment, with or without autistic features. This paper describes the case of a seven-year-old girl mainly presenting with autism spectrum disorder, language impairment, and intellectual disability. In addition, she also exhibited signs of attention deficit hyperactivity disorder. Whole-exome sequencing showed that she had a mutation in the FOXP1 gene; the variant revealed was FOXP1: NM_001244813 with a deleted segment (1152-1164) of exon 11. Subsequently, she was diagnosed with FOXP1 syndrome. In order to manage behavioral disturbance, risperidone was given, and she showed marked improvement. In this article, we report the characteristic features of attention deficits hyperactivity in addition to previously reported autism spectrum disorder with language impairment accompanied by intellectual disability caused by FOXP1 exon deletion. This study aims to provide a systematic, comprehensive presentation of a patient with a FOXP1 mutation to contribute to the existing literature on this subject.

## Introduction

Forkhead box protein 1 (FOXP1) (OMIM: 605515) is located at chromosomal region 3p14.1, which codes for a transcriptional repressor protein. FOXP1 is a member of the FOXP subfamily, which belongs to the FOX genome family. This family is a large and highly conservative family of transcription factors. The FOXP subfamily has four members, FOXP1-FOXP4, which maintain a high degree of conservatism within structures but also participate in various developmental processes, such as neural development related to language and cognition. The FOXP1 protein is widely expressed in human tissues and is involved in regulating the development of the brain, heart, lung, esophagus, immune system, and spinal motor neurons [1,2].

FOXP1 syndrome (FOXP1S) (OMIM #613670) is caused by FOXP1 gene deletions and mutations (nonsense, missense, and in-frame deletions). It is identified by the presence of intellectual disability with language impairment, with or without autistic features. Clinically, it may manifest as systemic developmental impairment; delays in walking; language impairment; and behavior disorders, including autism spectrum disorder (ASD), irritability, attention deficit hyperactivity disorder (ADHD), aggression, and stiff behavior [1,2].

To date, there have been more than 100 cases described in the literature [1,3]. We aim to provide a systematic, comprehensive presentation of a patient with the FOXP1 mutation to contribute to the existing literature on this subject. 

## Case presentation

History of presenting illness

We report a case of a seven-year-old girl who presented with intellectual disability, language impairment, and autistic features. She is a product of a full-term, uneventful, uncomplicated pregnancy. She was noted to have dysmorphic features and esotropia. Developmentally, her motor milestones were achieved mildly late. She started to walk at the age of 18 months but was not able to walk unsupported until she was 24 months old. 

Around this time, her parents noticed that she had poor eye contact and poor communication skills. By this time, she could only babble, unable to produce any comprehensible speech. Her first spoken word was at 36 months. She was unable to comprehend tasks and commands given to her. When she was three years old, she underwent a bilateral surgical correction of strabismus. Along with poor communication skills and poor eye contact, she exhibited repetitive patterns of behavior, interests, and activities. She constantly sucked her finger, flapped her hands, and nodded her head characteristically. She was particularly disturbed by loud noises and fixated on yellow lights. She enjoyed playing on the swing and arranging cubes. 

In terms of ADHD, she had a poor attention span and became distracted easily. She was always on the move, produced loud voices when playing, and had difficulty sitting on a chair without moving. On three different occasions, she left the house and was brought back by a neighbor. She was described to have a sensitive temperament. About a year ago, she was enrolled in a daycare center. Generally, a mild improvement was noted by both her therapists and parents. Her attention span and communication skills showed limited improvement. She is still suffering from a profound speech impairment. In terms of functioning, she is still unable to feed herself and still wears diapers. In addition, she could not complete toilet training.

Social and family history 

The patient was born to healthy, consanguineous parents (second-degree relatives). She had four siblings, two older brothers; and two younger sisters. All of them were healthy. She also had one cousin who suffered from ADHD. 

Physical examination 

The patient looked to be her stated age and had an average body build. She had dysmorphic facial features of frontal bossing, prominent occiput, small eyes with a downward-slanting palpebral fissure, and a bulbous nasal tip. In addition, she had a scar on her right upper forehead. She does not interact with the team and does not make any eye contact. She frequently sucked her thumbs. She had a labile affect, shifting between crying and laughing without apparent reason. She was unaware of her surroundings, impulsive and hyperactive, and threw temper tantrums. She babbled, used a strange repetitive speech, and did not produce any understandable speech. She seemed to have a good relationship with her father, mother, and oldest brother, who all seemed to be supportive. 

Laboratory tests and imaging 

Her laboratory tests showed results that were all within normal limits. Blood counts, renal and liver function tests, thyroid profile, and hemoglobin A1c (HbA1C) were unremarkable. An electroencephalogram (EEG) was performed and revealed nonspecific cerebral dysfunction and no epileptiform discharges. A brain magnetic resonance angiography (MRA) showed extensive prominent collateral venous vessels overlying cerebral convexities and deep venous system with non-visualization of the straight sinus and small-caliber transverse sinuses, bilaterally. This suggests the presence of a chronic venous occlusive disease. In addition, mild non-specific frontal white matter disease was detected. The patient’s echocardiogram showed normal cardiac structure and function. 

Genetic assessment 

Human whole-exome sequencing (WES) was performed and revealed a heterozygous FOXP1 gene variant that was classiﬁed as pathogenic according to the American College of Medical Genetics and Genomics (ACMG) guidelines. The variant revealed was FOXP1: NM_001244813 with a deleted segment (1152-1164) of exon 11. Then, an array comparative genomic hybridization (aCGH) was performed on the patient’s parents and revealed normal genes. Thus, the mutation was found to be spontaneous. 

Diagnosis 

As the patient was found to have FOXP1 mutation upon genetic testing, at the age of 4.7 years old, she was diagnosed with FOXP1 syndrome. She was found to meet the DSM-5 criteria for ASD, ADHD (combined-type), and intellectual disability (ID). She scored 43 on a Vineland adaptive behavior scale, suggesting a moderate delay in adaptive skills. Vanderbilt’s parents’ and teacher’s versions were completed too and showed eight out of nine symptoms of both inattentive and impulsive/hyperactive domains. The Bayley III scale revealed an age equivalent of two years old in the cognitive domain and fine motor, one-and-half years old in gross motor, and one-year-old in expressive and repetitive language development. A formal intelligence quotient (IQ) test could not be obtained, as the patient was unable to understand the concepts of the tasks, but based on developmental and adaptive testing, it was estimated to be SS= 40-50, placing her in the moderate intellectual disability range. 

Management 

For the past year, the patient has been going to a daycare center where she is being followed up by occupational, behavioral, and speech therapists. She has shown mild improvement in her attention span and communication skills. Despite dedicated work in behavioral and speech therapy, she is still showing dysfunctional behavior and profound language impairment. Just recently, during the last year, she has learned to name colors and numbers when she sees them in pictures. For initial pharmacological management, 0.25 mg of risperidone was prescribed, which was then increased to 0.5 mg. 

## Discussion

The association between FOXP1 and intellectual disability (ID) was first reported by Pariani and colleagues in 2009. A systematic review that evaluated the literature from then until July 2020 was carried out. The authors of this review identified 62 individuals with a FOXP1 mutation. Dysmorphic features were described in 59 cases, with the most common features being a prominent forehead, short nose with a broad tip or base, down-slanting palpebral fissures, ptosis, thick vermillion, ocular hypertelorism, and frontal hair upsweep [1]. All of these features were seen in this case. 

Speech and language delays were found in all cases; gross and fine motor delays were found in 97% of cases. Mild to moderate ID or global developmental delay was present in 90% of cases [1]. All of these elements were seen in this patient, as evident from IQ testing, Bayley III scale, and multidisciplinary team assessment. The child was able to walk unaided at 24 months, and her first spoken word came at 36 months. These parameters are closely related to the mean ages for walking unaided and speaking a first word found in the literature, which was 24.4 and 33 months, respectively [1]. 

About 57% of patients reported psychiatric comorbidities. ASD was found in 50% of cases. Other psychiatric comorbidities were ADHD, aggression, obsessive-compulsive traits, mood disorders, and anxiety [1]. This child showed symptoms of ASD and ADHD, along with aggressive behavior that was identified by the assessment tools mentioned previously. However, no other psychiatric diagnosis was identified.

Neurologically, hypertonia/muscle spasms, contractures, and seizures have been described in 20, 16, and seven individuals, respectively [1]. None of these were seen in this case. A total of 29 individuals had brain abnormalities evident from brain imaging, such as dilated lateral ventricles, white matter abnormalities, arachnoid cysts, large cisterna magna, corpus callosum defects, moderate frontal atrophy, cerebellar defects, and Chiari I malformation [1]. However, our patient showed normal brain imaging with no evidence of any structural damage. A structural heart abnormality was detected in 17 individuals, including patent ductus arteriosus, patent foramen ovale, pulmonary stenosis, and/or atrial septal defect [1]. In contrast, this patient had a normal cardiac structure and function. This patient also had strabismus that was corrected surgically. In addition to the 11 cases reported in the systematic review, two cases were reported later and showed strabismus [4,5].

In our case, along with behavioral therapy, we opted to use psychopharmacological intervention to help with irritability and aggressive and impulsive behaviors after behavioral interventions failed. The family agreed to consider the use of risperidone to help her current functional impairment and postpone her psychostimulant trial after stabilizing her dysfunctional behavior. The family was concerned about the side effects of the use of stimulants; however, they were still willing to consider it. A small dose of risperidone (0.25 mg) was used after a baseline metabolic screening workup was carried out following the Canadian Alliance for Monitoring Effectiveness and Safety of Antipsychotics in Children (CAMESA) guidelines [6]. The patient’s parents noticed the most improvement in her behavior on the fourth day, as well as her being calmer and more responsive. The treatment team and family are currently considering the implementation of other behavioral interventions as part of an individualized rehabilitation program. Given the complexity of this presentation, all newly approved interventions, such as digital health interventions, should be evaluated [7].

## Conclusions

This is the first case of this syndrome to be reported in Saudi Arabia. The high prevalence of both medical and mental health comorbidities, in this case, requires a multidisciplinary team approach and the use of standardized assessments and a diagnostic and statistical manual of mental disorders (DSM)-5-based assessment to establish the diagnosis and construct a multimodal intervention plan.
